# Artificial Intelligence: Help or Hindrance for Family Physicians?

**DOI:** 10.12669/pjms.37.1.3351

**Published:** 2021

**Authors:** Farhana Irfan

**Affiliations:** 1 Farhana Irfan King Saud University Chair for Medical Education Research and Development, Department of Family and Community Medicine, College of Medicine, King Saud University, Riyadh, Saudi Arabia

**Keywords:** Artificial Intelligence, Family Physicians, Machine learning

## Abstract

The use of Artificial Intelligence (AI) and related technologies is rapidly increasing and its application in clinical practice is a promising area of development. Artificial Intelligence can be a solution in the future as a physician’s new assistant; AI-physician combinations can act like models of ‘peaceful co-existence’. While it has the potential to mold many dimensions of patient care and can augment quality improvement, it cannot replace a family physician’s diagnostic intelligence, empathy and relationships. Physicians need to strike a balance between these combinations for better health outcomes without increasing patients’ frustration.

The use of Artificial Intelligence (AI) and related technologies is rapidly increasing. What is Artificial Intelligence? It is not one technology, but rather a collection of techniques that allow computers to work and react like humans by problem-solving. Its use is prevalent in almost all fields and is beginning to be applied in healthcare too^1^. AI has the potential to mold many dimensions of patient care but we have yet to grasp fully the speed and breadth of this new revolution. At present, AI is being utilized in the medical field by being incorporated into health systems; varying from simple scheduling of appointments, online medical records, reminders for follow-ups to diagnosis in the fields of radiology, pathology, and ophthalmology 2,3. The field of Family Medicine is also evolving rapidly to meet growing patient demands such as catering to patients of all age groups with chronic or acute illnesses, or both. In general, the family physicians are the first to see patients, when the illness is still in the process of development. They are expected to diagnose individuals earlier on and avoid excessive testing and referrals^4^,^5^. Currently, a major amount of a doctor’s time is spent on computer work or on other administrative tasks, instead of with patients^6^. The “art” of Family medicine is being lost. Physicians feel increasingly stuck with desk work. Patients are not “looked at” anymore, giving a feeling that doctors are obsessively fixated on information, charts and monitors. This has led to erosion in the doctor-patient relationship over recent years. With increasing life expectancies, the patient load will further increase and so will the computer work in the coming years. The question arises; how we can give more time to patients without compromising the quality and patient care? Artificial Intelligence can be a solution in the future as a physician’s new assistant.

In healthcare, AI studies suggest productivity gains^7^,^8^ and there is a growing interest in using AI to augment and improve the practice globally. However, there is limited literature available on its use in low resource settings where its application is nascent and its voice needs to be amplified to shape its future. Developing countries grapple with high burden of disease as well as with the poor healthcare infrastructure. A local survey on the use of AI in healthcare in low resource settings reported lack of trained AI professionals as the most common (64%) hurdle followed by difficulty in identifying use cases (28%). It’s no surprise that the medical community might not be familiar with its current applications possible in the country. A PubMed search revealed AI publications in the field of medical education^9^ and across various specialties of medicine^10^-^13^ but none in the discipline of Family Medicine locally.

The degree to which AI can improve primary care is undetermined, a review of its application at the primary care level will accelerate realizing of the full potential for improving the healthcare thus making it achievable.

There are significant hypothesis on the capacity of AI, which are still developing. The main purpose can be improving processes and relieving some of the physician’s workload. Additionally, the possible areas that can be covered are; improved quality of care on routinely collected administrative data (such as triaging patient inquiries and processing patient claims) and assistance for physicians by providing up-to-date medical information from journals, , textbooks and clinical practices, so that they can be informed on proper patient care.^14^,^15^

Clinical documentation is becoming mandatory worldwide and consumes a lot of time on a daily basis. Taking patient histories and making decisions with a standard computer is time consuming and distracts physicians from paying attention to the patient. The data that is obtained towards the end of consultation is either dismissed or ignored. As a consequence, GPs may unfortunately produce a misrepresented document with a distorted range of differential diagnoses.^16^ Unlike physicians, AI systems never get tired or irritable. Machine learning can be used for natural language processing, speech recognition and text analysis. It can transcribe patient interactions, analyze unstructured clinical notes on patients and give a probable diagnosis. It could assist GPs in recognizing important aspects and overcoming cognitive biases, thus guiding about tests and reasonable treatment options 17-20 Clinicians can discuss more with the patients if AI takes over the mundane data-entry tasks.^21^

In addition, symptom checkers can serve in helping educate patients on the range of diagnoses that might fit their symptoms. The triage function informs patients whether they should seek care at all and, if so, where and with what urgency, empowering the patients with self-treatable conditions rather than feeling dependent on their physicians^22^. Audit of vignette based symptom checkers has shown to provide appropriate advice for up to 80% for emergency cases^23^. It can also screen patients, supplement the triage lines, manage a preliminary analysis suggesting likely diagnosis (thus reducing the number of visits, which can save patients’ time and money) and in addition, may decrease demand on primary care providers.^1^,^4^,^14^,^16^-^18^,^20^

AI can have a considerable role in preventative medicine by summarizing a large set of medical information and using algorithms to proactively propose consultations, suggesting optimal treatments. It can also bring specialist skills into primary care by early diagnosis of diseases and recognition of high risk patients by pattern recognition in imaging results such as automated diagnosis of diabetic retinopathy, interpreting radiographs and ultrasound.^24^-^26^ Relevant patient education material and reminders can be effective in personalizing care and can be very promising in the future. It can also be a good option to support large under resourced populations where human expertise is scarce.^26^-^28^

It is often seen that communication skills are a major reason for patient dissatisfaction. There is concern that these skills remain basic even after training. There is evidence that communications skills can be effectively developed but lack of feedback on performance is an obstacle for improvement; primarily as it is time consuming for physicians. AI has a prospective role for assessing and training physician’s communication skills by analyzing the recorded consultation and providing feedback on the delivery such as proportion of talk by patient and clinician, overlapping talk, speed of speech, tone of voice and language, clinical jargons etc. for improvement.^17^

Furthermore, what does AI mean to general practitioners and for primary care? It does have the potential to bring significant change to healthcare but, there is contemplation on the risks too. Like any other innovation, it requires careful thought. AI has a significant amount of hype associated with it, which needs to be evaluated before complete adoption. This matter requires discussion. Can AI replace clinical experience and human decision? Can AI have the sixth sense that physicians feel in challenging cases? Can the machines understand the family medicine patient care context? Can it replace a doctor’s clinical diligence? There will always be the lingering threat of over or under diagnoses. All this requires having humans (physicians) at the center.

These applications are designed to amplify human cognition and work, rather than replace it; they can be better calculators but not better thinkers. AI-physician combinations should act like models of ‘peaceful co-existence’, like autopilots on planes; improved safety without compromising the training of pilots^29^. It can free physicians from spadework and let them focus on the humanistic elements of care and practice in line with the roots of medicine as a healing profession. Another obstacle is that it has to be tuned to the correct data, from the appropriate population. AI diagnostic tools developed in one country are not applicable worldwide and will not provide the correct guidance to a primary care setting elsewhere, as one size does not fit all.

At this moment, the physicians lack the expertise required to use AI in family practice. This raises an important question about the sufficiency of medical curricula to equip future physicians for the advancement in clinical practice. Medical schools need to incorporate courses on AI in medical school curriculum to increase the awareness as well as highlight the importance of AI during undergraduate as well as postgraduate training in medical education^9^ as improvements in education, may improve the rift. Family medicine training programs need to deploy appropriate training and educational programs for physicians on how to use data science and AI for better services. Family practice can embrace it and evaluate it as any new medical innovation and avail the scope it offers. Physicians need to strike a balance between these combinations for better health outcomes without increasing the frustration of patients.

This is a preliminary descriptive analysis which provides insights into the ways technological advancements can impact the primary care, the perceived limitations and benefits. This must be read with a futuristic and contemporary vision as a bridge to fill in the gaps in the fragmented knowledge that have been elucidated. If it is rightly and timely adopted it can help physicians to achieve better patient care.

To conclude, AI can augment quality improvements in health care at reduced costs, but it cannot replace a family physician’s diagnostic intelligence, empathy and relationships^2^. The question arises; can AI incorporate and respond to visual clues and personal preferences, maintain patient dignity and cultural and social demands, while also overcoming undocumented, immeasurable events and ethical concerns? Physicians will remain important and should become tech savvy to work side by side with it; as the patient population will continue to grow in the foreseeable future.
